# Fluorescence Anisotropy Sensor Comprising a Dual Hollow-Core Antiresonant Fiber Polarization Beam Splitter

**DOI:** 10.3390/s20113321

**Published:** 2020-06-11

**Authors:** Hanna Izabela Stawska, Maciej Andrzej Popenda

**Affiliations:** Department of Telecommunications and Teleinformatics, Wroclaw University of Science and Technology, 50-370 Wroclaw, Poland; maciej.popenda@pwr.edu.pl

**Keywords:** microstructured fibers, hollow-core fibers, dual hollow-core fibers, antiresonant fibers, optical fiber design, optical fiber sensors, multiphoton fluorescence, fluorescence anisotropy

## Abstract

Fluorescence anisotropy imaging and sensing is a widely recognized method for studying molecular orientation and mobility. However, introducing this technique to in vivo systems is a challenging task, especially when one considers multiphoton excitation methods. Past two decades have brought a possible solution to this issue in the form of hollow-core antiresonant fibers (HC-ARFs). The continuous development of their fabrication technology has resulted in the appearance of more and more sophisticated structures. One of the most promising concepts concerns dual hollow-core antiresonant fibers (DHC-ARFs), which can be used to split and combine optical signals, effectively working as optical fiber couplers. In this paper, the design of a fluorescence anisotropy sensor based on a DHC-ARF structure is presented. The main purpose of the proposed DHC-ARF is multiphoton-excited fluorescence spectroscopy; however, other applications are also possible.

## 1. Introduction

Hollow core antiresonant fibers (HCARFs) attract a great deal of researchers’ attention due to their remarkable optical features such as several transmission bands over which they exhibit not only low losses, but also low nonlinearities and low dispersion [1]. The propagation of light along these fibers can be described by the well-known ARROW (Anti-Resonant Reflecting Optical Waveguide) model [2,3]:

(1) $$\lambda_{k}=4d_{0}\frac{\sqrt{n_{g}{}^{2}-1}}{\left(2k+1\right)}$$

where *λ_k_* is the wavelength of the considered transmission window, *d_0_* is the thickness of the first layer of the high-index material surrounding the core, *n_g_* is the material’s refractive index and *k* is the number of the transmission window (0,1,2,…). To take full advantage of their properties, several research groups have begun elaborating other hollow-core fiber-integrated optical devices, such as optical fiber couplers [4,5], filters [6] or sensors [7,8], including multiphoton fluorescence optical fiber sensor setups [9,10]. The latter is a very promising area and is already delivering some remarkable results in biological and medical research by employing not only multiphoton-excited fluorescence but also other non-linear phenomena such as, for example, second harmonic generation or coherent anti-stokes Raman scattering [11,12]. However, HCARF-based sensors employing non-linear methods for medical diagnostics up to this day lack one interesting functionality—namely the capability of measuring fluorescence anisotropy.

Fluorescence anisotropy (FA) measurements are a powerful optical technique based on measurements of the signal’s polarization. By investigating the polarization properties of the fluorescence one can obtain additional molecular information about the investigated biological sample, such as the molecular orientation, aggregation [13,14], rotational diffusion [15], or energy migration (homo-FRET) [16]. The principle of FA is based on the fact that upon excitation with polarized light the emission from many samples is also polarized. Assuming that a sample is illuminated by horizontally polarized light, the intensities of the fluorescence signals observed through a vertically or horizontally oriented polarizer are usually uneven. The origin of this phenomenon can be found in the existence of transition moments that align in a specific direction within a fluorophore [17]. The depolarization of the fluorescence signal can occur due to several factors, namely the non-parallel absorption and emission dipole moment of the fluorophore, its fast rotational movement or non-radiative energy transfer to another fluorophore (FRET) [18]. FA is also known to possess, similarly to the fluorescence lifetime, the “intensive property”, which means that is independent of the amount of material [19,20] and offers the ability of quantitative measurement without the separation step, i.e., without removing one of the components from the solution. This feature makes FA useful in studying protein-ligand and protein-protein interactions [16,19,21,22,23,24], as well as in the diagnostics of cancerous tissue [25,26,27].

The probability of the one-photon excitation (1PE) of the molecule is proportional to cos^2^*θ*, where *θ* is the angle between the transition moment of the molecule and the vertical axis. Assuming the absence of depolarizing processes, the value of the fundamental anisotropy *r_f_* can be determined using equation [17]:

(2) $$r_{f}=0.6\left(\cos^{2}\beta -1/3\right)$$

where *β* is an angle between absorption and emission transition moments. Fundamental anisotropy in this case is often within the range [28]:

(3) $$-0.2\leq r_{f}\leq 0.4$$

It is worth noting that non-linear, multiphoton absorption methods can positively influence the specificity of FA. Apart from the well-known benefits of using longer wavelengths, such as reduced scattering by the sample, lower phototoxicity etc., anisotropy is also known to be influenced directly by the mode of excitation. Indeed, in the case of two photon excitation (2PE), *r_f_* is 4/7 due to a higher photoselection for the two-photon absorption process [29]; above is a result of the 2PE’s probability being proportional to cos^4^*θ* instead of cos^2^*θ*, as in the case of 1PE [29,30,31]. Similar results have been observed for the three- and four-photon excitation, which follow the cos^6^*θ* and cos^8^*θ* photoselection rules and have their *r_f_* equal to 2/3 and 8/11, respectively [20,32,33,34].

**Figure 1:**
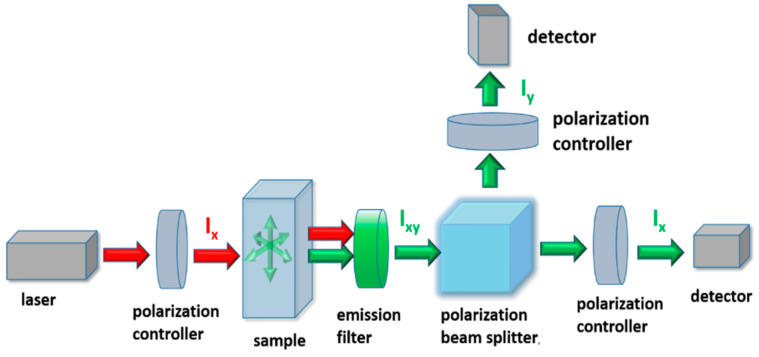
Typical setup for the fluorescence anisotropy measurement in a two channel configuration. The linear polarized excitation signal from the laser lights the sample. The fluorescence signal is selected by an emission filter, then two orthogonal states of polarization are separated using a polarization beam splitter and are measured simultaneously using two detectors.

In practice, if the excitation light is vertically polarized (along the *Y*-axis), the fluorescence anisotropy *r* can be determined by a simple ratio of signals with different polarizations [17]:

(4) $$r=\frac{I_{y}-GI_{x}}{I_{y}+2GI_{x}}\;$$

where *G = I_y_/I_x_* is a correction factor introduced to compensate the transmission efficiencies of the optics for the two polarizations. In general, two optical setups can be distinguished for FA measurements [20,22] (Figure 1). The first—single channel method—uses a single photodetector to measure the emitted fluorescence, making it necessary to perform two measurements to obtain the intensities of the horizontal (*I_x_*) and vertical (*I_y_*) polarized signals. The second, known as dual-channel configuration, employs two photodetectors, one for each of the polarizations, allowing for their simultaneous observation. These anisotropy detection schemes are often integrated with fluorescence microscopy systems. However, performing FA measurements in an endoscopic manner is a challenging task. Up to this moment, only a few solutions of FA measurement setups with an in-vivo potential have been presented [35,36,37]. These methods require special calibration schemes in order to compensate for depolarization factors such as telescope optics. Additionally, diameters of such endoscopes are usually in the millimeter region, making the examination procedure rather invasive. Overall, up to this date fiber optic-based anisotropy setups are still rather uncommon. The level of setup complexity arises even more if one considers exciting fluorescence in a non-linear manner, while still using optical fibers. Material dispersion and nonlinearities can severely distort the excitation signal propagating through the fiber and make the multiphoton absorption process very inefficient. Both of those problems can easily be overcome by employing HCARFs’ however, those fibers do not allow to maintain the polarization of the guided signal, and hence do not provide the means necessary to conduct FA measurements. A potential optical device which could allow one to perform multiphoton FA in a completely fiberized manner would be a dual hollow-core antiresonant fiber (DHC-ARF). The idea of splitting signals with different polarizations using DHC-ARF structures has only been analyzed theoretically so far in terms of their optical and geometrical properties [38,39]. However, according to the authors’ best knowledge, no results have been reported where a DHC-ARF (or any other kind of dual-core fiber) was used in a fluorescence anisotropy detection setup, with the fiber being the main part of the sensor. Such a sensor could potentially be comprised of a single optical fiber, allowing for a significant reduction in size and an increased efficiency of the multiphoton fluorescence excitation.

In this paper, we present a theoretical investigation of a DHC-ARF-based fluorescence anisotropy sensor. Based on our experience from previous work [39], we propose a DHC-ARF with a structure comprising only circular-shaped capillaries. The structure has been designed to allow for an efficient transmission of signals from the NIR (Near-Infrared) and VIS (Visible) wavelengths region, hence being a viable solution for multiphoton excited fluorescence experiments. Additionally, its polarization-splitting parameters have been analyzed, allowing to determine its possible use in a multiphoton excited fluorescence anisotropy setup. 

## 2. Polarization Beam Splitter

Using a small segment of a dual core fiber (DCF) is a known method for preparing a polarization beam splitter [38]. According to the coupled mode theory, DCF supports four supermodes: two even and two odd. In order to turn a DCF into a polarization beam splitter, several optical parameters should be considered, out of which the most important are the coupling length ratio (*CLR*) and the coupling length (*L_c_*), defined by the following expressions [5]:

(5) $$CLR=\frac{L_{c}^{x}}{L_{c}^{y}}\;$$

(6) $$L_{c}^{i}=\frac{\pi }{\beta_{even}^{i}-\beta_{odd}^{i}}\;$$

where *i* denotes the linear polarization direction (either *X* or *Y*), while *β_even_* and *β_odd_* are propagation constants of the even and odd supermodes, respectively. In the case of a polarization beam splitter, the value of *CLR* should be close to 2 $(L_{c}^{X}$ > $L_{c}^{Y})$ or 0.5 $(L_{c}^{X}$ < $L_{c}^{Y})$, ensuring that the corresponding power transfer maxima for signals with different polarizations will be shifted in phase by *π/2* and signals with orthogonal polarization states will be split between the cores of the fiber [40]. 

Other parameters which should be considered are the extinction ratio (*ER*) and high-order mode extinction ratio (*HOMER*). The former can be defined as the normalized power ratio between the *X*- and *Y*-polarized signals in the same core:

(7) $$ER=10\log_{10}\left(\frac{P_{j}^{X}}{P_{j}^{Y}}\right)$$

where $P_{j}^{X}$ and $P_{j}^{Y}$ are the powers of the *X*- and *Y*-polarized signals in the *j-th* core (*j =* A or B), while *HOMER* is the ratio of the lowest high-order mode loss to the highest fundamental mode loss:

(8) $$HOMER=\frac{\alpha_{HOM}}{\alpha_{FM}}$$

where *α_HOM_* and *α_FM_* are the attenuations (in dB/m) of the high-order and fundamental modes, respectively.

Designing a DHC-ARF polarization beam splitter for fluorescence anisotropy measurements started by ensuring its proper transmission bands—one in the 800–1300 nm range and the other near 560 nm. The first window covers the wavelengths commonly used for both two- and three-photon excitation. What is more, the wavelength range from 600–1350 nm is the so called therapeutic window where the absorption of the tissue is limited [41]. Additionally, the availability of the ultrafast light sources emitting at these wavelengths is also very good. The choice of 560 nm was due to the fact that it is near the emission peak of flavin adenine dinucleotide (FAD), an important endogenous fluorophore [42]. Eventually, two main wavelengths were chosen—the excitation wavelength, *λ_ex_*, equal to 1064 nm and the fluorescence wavelength, *λ_fluo_*, equal to 560 nm.

**Figure 2:**
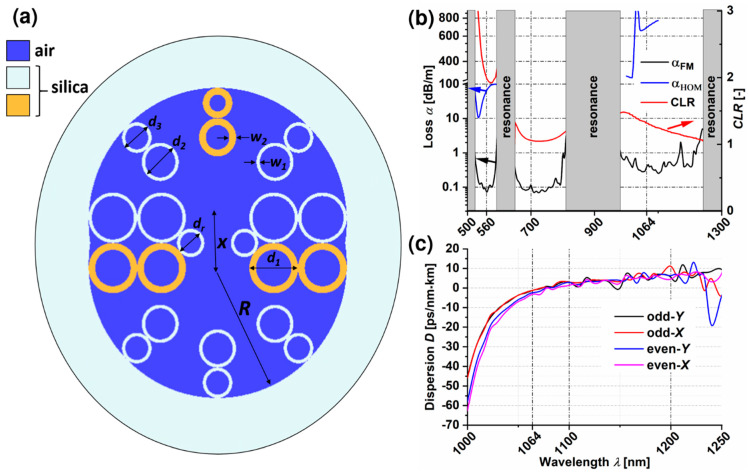
(**a**) Cross section of the DHC-ARF; the descriptions and values of the dimensions can be found in the main text. The cross-section is rotated by 90° when compared to the one presented in Figure 3. (**b**) The highest losses of the fundamental modes (α_FM_, black curve), the lowest losses of the higher order modes (α_HOM_, blue curve) and the coupling length ratio (*CLR*, red curve) with respect to the wavelength. The arrows indicate the *Y*-axis corresponding to the pointed curve. (**c**) Dispersion *D* versus wavelength of the four fundamental core modes with different orthogonal polarizations (-*Y* or *–X*) and parity (even or odd) in the 3rd antiresonant window of the proposed DHC-ARF. For *λ_ex_* = 1064 nm one can see that *D* ϵ < 0, 5 > ps/nm × km, making it a negligible value for the multiphoton fluorescence-based diagnostic setups.

The structure of the previously presented DHCARF is rather complicated due to the presence of elliptical capillaries [39]. HCARFs with such capillaries have not been fabricated up to this date, which can be a result of two factors. First, elliptical capillaries can be considered an uncommon, custom-made material [43]. Second, and even more important, are the general problems that occur during the fiber drawing procedure, connected with maintaining the elliptical shape of the capillaries [44]. On the other hand, more and more sophisticated structures are both being manufactured [45] and analyzed [46,47,48], including a dual-ring cladding HCARF [49]. As a result, the decision was made to re-design the structure of the DHC-ARF and replace the elliptical capillaries with circular ones, as presented in Figure 2a. The inner radius of the tubes which form the solid cladding of the fiber is *R* = 35 µm, while the distance between the centers of those tubes is *x* = 12.5 µm. The diameters of the capillaries of the antiresonant structure are *d_1_* = 13.2 µm, *d_2_* = 10 µm, *d_3_* = 8 µm and *d_r_ =* 7.4 µm. In order to control the optical properties of the DHC-ARF (loss and *CLR*), the thicknesses of the core-surrounding capillaries—*w_1_* and *w_2_*—should be carefully selected. The value of *w_1_* was calculated directly from Equation (1), while *w_2_* was swept for different values in order to obtain proper *CLR*. The complete procedure of using *w_1_* and *w_2_* (as well as the other geometrical parameters) to modify the DHC-ARF’s optical parameters is described in detail in [39]. In short, two types of capillaries (thick and thin ones, described by *w_1_* and *w_2_*, respectively) have effectively made the proposed structure birefringent. Additionally, those capillaries were placed asymmetrically for each core (i.e., they are oriented along different directions, perpendicular to each other; see, for example, the orange-colored ones in Figure 2a), which allowed us to control the *CLR* by varying the value of *w_2_*. Eventually, *w_1_* and *w_2_* were found to be 900 nm and 242 nm, respectively; the value of *w_2_* was then slightly changed to 240 nm in order to maintain a more realistic value in terms of manufacturing the structure.

**Figure 3:**
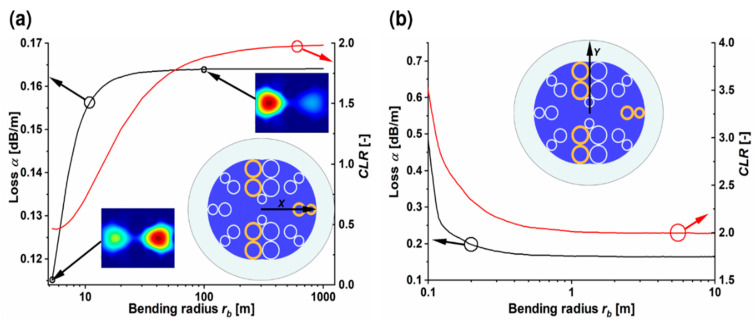
Bending properties of the DHC-ARF. Loss and *CLR* coefficient for different bending radii along (**a**) the *X*-axis and (**b**) *Y*-axis. The insets present the cross section of the DHC-ARF together with the black arrows indicating the orientation of the bending direction (marked as *X* or *Y* in the cross-section of the structure). Additionally, in (**a**) two mode profiles at *r_b_* = 5 m and 100 m are presented, showing the energy transfer between the cores as the bending increases. The red and black arrows, connected with the circles of the same color, indicate the *Y*-axis corresponding to the circled curve.

Lumerical^®^ Mode Solutions commercial software [50] was used to calculate the optical parameters of the DHC-ARF; the optical properties of glass were modeled by means of the Palik model, contained in the Lumerical^®^ material library. The simulation mesh basic element was a 20 × 20 nm square, with PML (Perfectly Matched Layer) boundary conditions. The dependences of the loss (α) of the fundamental mode (FM), the high order mode (HOM) and *CLR* on the wavelength are presented in Figure 2b. For the sake of clarity, only the highest-loss FM and the lowest-loss HOM are presented. Three transmission windows can be distinguished, namely 520–590, 650–807 and 970–1233 nm, which is consistent with Equation (1). The first transmission window covers the fluorescence emission spectrum, while the second and third include wavelengths which are often used for the excitation purposes of multiphoton fluorescence. Losses of the FM (black line) at *λ_fluo_* and *λ_ex_* are α_FM560_ ≈ 0.07 dB/m and α_FM1064_ ≈ 0.4 dB/m, respectively. Due to the long and resource-demanding calculations, the HOMs’ losses (blue curve) were calculated only in the vicinity of the wavelengths of interest, namely *λ_fluo_* and *λ_ex_*. Thus, for 560 nm α_HOM560_ ≈ 65 dB/m and for 1064 nm α_HOM1064_ ≈ 700 dB/m. In Figure 2b, the relationship between *CLR* and wavelength is also presented (red curve). This characteristic is most important in the first transmission window where the polarization-dependent splitting of the fluorescence signal will occur. The change in the values of *CLR* in this region of wavelength is large and only for a relatively narrow range *CLR* is close to 2. Specifically, 1.9 < *CLR* < 2.1 for 550 nm < *λ* < 590 nm, with *CLR* = 2 for *λ_fluo_*, showing that the structure could possibly allow to split green-fluorescence signals according to their polarization. Additionally, as was mentioned in the introduction, the dispersion of the glass is one of the main factors limiting the use of optical fibers in non-linear experiments and hollow-core fibers are capable of circumventing this problem. Indeed, dispersion versus wavelength dependences of the DHC-ARF’s four fundamental modes, presented in Figure 2c, prove that the proposed DHC-ARF maintains one of the main features of HCARFs—a very low dispersion across the whole width of the transmission window. According to the conducted calculations, excitation impulses at *λ_ex_* should be only slightly affected by the fiber’s dispersion, which is −3.1 ps/nm × km in the worst case of the even-*X* fundamental mode. Since HCARFs (especially the single-ring ones) are known to be susceptible to bending, an analysis of α and *CLR* versus bending radius was also performed (Figure 3). The calculations were conducted for *λ_fluo_*, for both *X* and *Y* polarization directions. As it is shown, *CLR* is very sensitive to bending, especially if its direction lies along the *X* axis which is oriented also along the coupling channel. In this case the fiber should be as straight as possible (*r_b_* > 800 m) to keep the *CLR* in the range of <1.9, 2>. The loss of the fiber is less sensitive and a significant influence of bending can be noticed when *r_b_* < 20 m. Nevertheless, they are being kept at a reasonable level of α < 0.17 dB/m. An interesting observation is that the smaller the *r_b_*, the smaller the α_FM_, while one would expect the exact opposite. This phenomenon can be explained by a different distribution of the electric field energy in the cores during bending in the *X* direction. In the insets of Figure 3a mode profiles are presented for two distinct bending radii, namely *r_b_* = 100 m and *r_b_* = 5 m. In the case of *r_b_* = 100 m, the mode energy is contained mainly in the left core. This core has two capillaries with thicknesses *w_2_* which do not exactly fulfill the antiresonant condition from Equation (1). Hence, the loss is slightly bigger than for *r_b_* = 5 m at which almost the whole energy is concentrated in the right core, where only one capillary with *w_2_* is present.

## 3. DHC-ARF-Based Fluorescence Anisotropy Sensor

**Figure 4:**
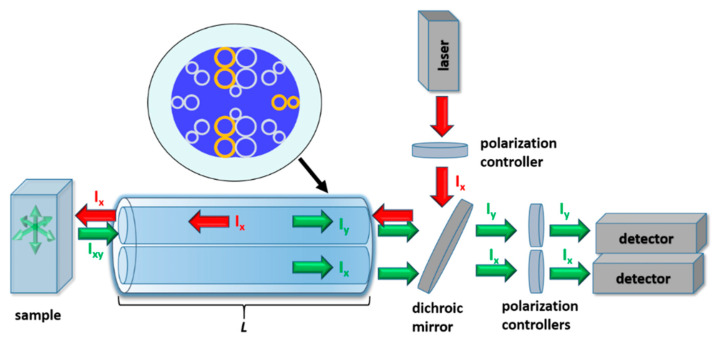
Fluorescence anisotropy measurement setup based on a DHC-ARF with length *L*. The excitation beam (laser) is coupled into one of the cores (excitation core) of the DHC-ARF. After the sample is excited, its fluorescence signal is collected via the same core. During the back-propagation of fluorescence, its two orthogonal polarization states are separated (one in each core of the DHC-ARF). Finally, their intensities are measured using two detectors. The presence of polarization controllers in front of the detectors allows the user to eliminate any signal with an unwanted state of polarization.

**Figure 5:**
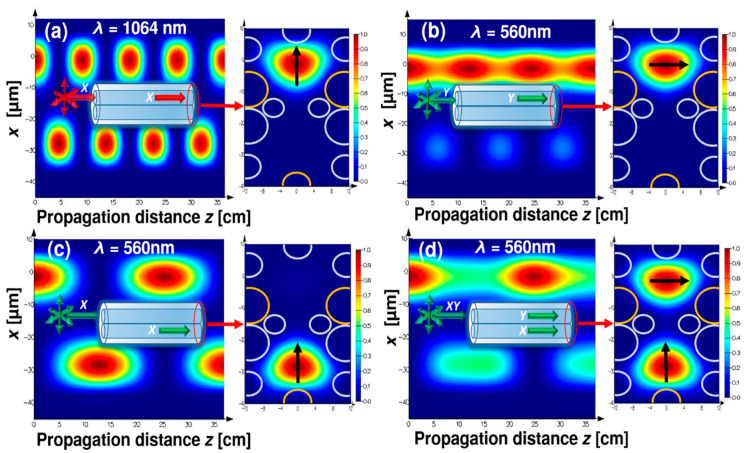
Distribution of the electric fields intensities during propagation along the DHC-ARF and at the output end of the DHC-ARF, for signals with different wavelengths and polarizations. Propagation of the (**a**) *X*-polarized excitation signal at 1064 nm, (**b**) *Y*-polarized fluorescence signal at 560 nm, (**c**) *X*-polarized fluorescence signal at 560 nm and (**d**) unpolarized fluorescence signal at 560 nm. Insets in (**a**–**d**) visualize the direction of propagation in the cores of DHCARF for a given situation; letters *X*- and *Y*- are related to the polarization direction. Red circles indicate the distal end of the DHC-ARF, where the output mode fields were calculated. Those fields are presented on the right of their corresponding propagation graphs, pointed out by the red arrows. The yellow and grey circles in the output mode field figures are the DHC-ARF’s capillaries, with different colors indicating capillaries with different thicknesses, as previously described in Figure 2a. Black arrows indicate the polarization direction of the output signal.

In Figure 4 an optical setup in which the DHC-ARF can possibly be used as a fluorescence anisotropy sensor is presented. The excitation light from the laser is coupled into one of the cores of the DHC-ARF. The length of the fiber is denoted as *L*. This core (further named as the excitation core) is also used for the purpose of fluorescence collection, while the other one is closed to prevent any unnecessary distortions of the signal, resulting from the fluorescence signal coupling between the cores. The fluorescence signal is transmitted along the fiber and, due to the polarization splitting capabilities of the proposed DHC-ARF, its two orthogonal polarization states can be distinguished and detected, each by a single detector. In order to deliver a linearly polarized excitation signal to thesample and separate two orthogonal polarization states of the collected fluorescence signal, *L* should be equal or very close to an integer multiple of *L_c_* calculated for 560 nm and 1064 nm wavelengths. For 560 nm, $L_{c}^{X}$ and $L_{c}^{Y}$ are ~12.7 and ~6.37 cm, respectively; for 1064 nm only $L_{c}^{X}$ was calculated, and its value is 4.49 cm. Based on those results and the fact that bending of the DHC-ARF should be avoided, *L* = 36.9 cm was chosen. In Figure 5, the propagation of *λ_ex_* and *λ_fluo_* signals along the length *L* of the DHC-ARF are shown. These simulations were conducted by means of the eigenmode expansion method implemented in the Lumerical^®^ Mode Solutions software. During these simulations we assumed that the excitation signal is introduced to the top core of the fiber, as shown in the inset of Figure 5a. The *X*-axis is oriented along the coupling channel, perpendicular to the fiber’s main axis. It can be noticed that after propagating the distance *L*, the state of polarization of the excitation signal is preserved and almost the whole energy is still located in the top (i.e., launch) core. In case of the propagation of the fluorescence signal, three different polarization cases were analyzed: *Y*-polarized (Figure 5b), *X*-polarized (Figure 5c) and unpolarized (Figure 5d), with the latter being simulated as a sum of the *X* and *Y* polarized signals with identical intensities. The intensity distributions at the distal end of DHC-ARF for Figure 5a–d are presented on the right of their corresponding propagation graphs. Perhaps the most interesting and important observations come from the results presented in Figure 5d, which show that even for an unpolarized signal (or simply a signal with an undetermined polarization) the division of the two orthogonal polarization states between the DHC-ARF cores is possible in a clean, 50–50 manner. In the case of the linear polarized fluorescence signals (*X* or *Y*), top core is used to detect *Y*-polarized light and bottom core to detect *X*-polarized light. Those results can be considered as supporting the ones presented in Figure 5d, since they show that after propagating the length *L* of the DHC-ARF, one can clearly distinguish between two different polarization states of the fluorescence signal. A more complete characterization of the DHC-ARF was ensured by calculating the values of *HOMER* and *ER* after the propagation length *L*. According to Equation (7), the values of *ER* for *X*- and *Y*- polarized light at 560 nm were found to be equal to 40 dB and 64 dB, respectively. These values further support the possibility of using the proposed DHC-ARF as a polarization beam splitter. Based on the previously calculated values of α_HOM_ and α_FM_ and using Equation (8), the value of *HOMER* for *λ_fluo_* is equal to 929. Hence, in order to obtain single mode guidance it is enough to use a segment of the fiber approximately 37 cm long. In such a case, the attenuation of the HOM will be close to 24 dB, which is sufficient for FA measurements.

## 4. Conclusions

In this paper, a new fluorescence sensor design based on the dual hollow-core, antiresonant fiber was presented. The fiber’s structure contains only circular-shaped capillaries in order to simplify the possible fabrication process. Important optical parameters like transmission windows, loss, bending loss, *CLR*, *HOMER* and *ER* were calculated. During the simulations three transmission windows in the VIS-NIR range of the proposed fiber were determined, namely 520–590, 650–807 and 970–1233 nm. Those windows make the fiber a possible candidate for a single-fiber, multiphoton-excited fluorescence sensor. Additionally, not only does the proposed fiber provide access to interesting (from the fluorescence experiments point of view) spectral windows, but it also delivers its own unique feature—the possibility of splitting signals with different states of polarization. The fiber’s *CLR* calculated at 560 nm is 2, allowing for an equal, 50–50 split of an unpolarized beam between both the fiber’s cores. The *ER* for the two linear polarization states (*X*- and *Y*-) is 40 and 64 dB, respectively, while *HOMER* is as high as 929. What is more, the presented fiber introduces only a minimal amount of dispersion (*D* ≈ −3.1 ps/nm × km, worst case) for the fundamental fiber modes, ensuring that a negligible amount of temporal distortion of ultrashort pulses of λ = 1064 nm will be introduced, hence increasing the multiphoton excitation efficiency. As a result, the presented DHC-ARF becomes an interesting idea of an optical fiber-based, multiphoton-excited fluorescence anisotropy sensor, with a potential future in in-vivo applications. The weakest feature of this fiber is its high sensitivity to bending. The results have shown that when bending along the *X*-axis, even for a very large bending radii (*r_b_* ≥ 100 m) the value of the *CLR* changes significantly, making it increasingly harder to distinguish between the different states of polarization between the fiber’s cores. However, since the predicted length of the fiber is approximately 36.9 cm, one can imagine preparing a rigid and stiff, needle-like fiber sensor head, which could possibly penetrate tissue in a minimally invasive manner and without the undesired, bending-related optical signal distortions.
